# RNA Primer Extension Hinders DNA Synthesis by *Escherichia coli* Mutagenic DNA Polymerase IV

**DOI:** 10.3389/fmicb.2017.00288

**Published:** 2017-03-01

**Authors:** Tommy F. Tashjian, Ida Lin, Verena Belt, Tiziana M. Cafarelli, Veronica G. Godoy

**Affiliations:** Godoy Lab, Department of Biology, Northeastern UniversityBoston, MA, USA

**Keywords:** DNA polymerase IV, DinB, RecA, protein–protein interactions, DNA replication

## Abstract

In *Escherichia coli* the highly conserved DNA damage regulated *dinB* gene encodes DNA Polymerase IV (DinB), an error prone specialized DNA polymerase with a central role in stress-induced mutagenesis. Since DinB is the DNA polymerase with the highest intracellular concentrations upon induction of the SOS response, further regulation must exist to maintain genomic stability. Remarkably, we find that DinB DNA synthesis is inherently poor when using an RNA primer compared to a DNA primer, while high fidelity DNA polymerases are known to have no primer preference. Moreover, we show that the poor DNA synthesis from an RNA primer is conserved in DNA polymerase Kappa, the human DinB homolog. The activity of DinB is modulated by interactions with several other proteins, one of which is the equally evolutionarily conserved recombinase RecA. This interaction is known to positively affect DinB’s fidelity on damaged templates. We find that upon interaction with RecA, DinB shows a significant reduction in DNA synthesis when using an RNA primer. Furthermore, with DinB or DinB:RecA a robust pause, sequence and lesion independent, occurs only when RNA is used as a primer. The robust pause is likely to result in abortive DNA synthesis when RNA is the primer. These data suggest a novel mechanism to prevent DinB synthesis when it is not needed despite its high concentrations, thus protecting genome stability.

## Introduction

The SOS gene network in *Escherichia coli* is a highly conserved global stress response induced by DNA damage caused by either exogenous sources or byproducts of cellular metabolism (Horii et al., 1981; Goodman and Woodgate, 2013). The SOS response is upregulated when single-stranded DNA (ssDNA), the signal of DNA damage, accumulates and is bound by the protein RecA. The interaction between RecA and ssDNA results in a new activity for the RecA nucleoprotein filament or RecA^∗^. The newly acquired co-protease activity of RecA^∗^ promotes the autocleavage of LexA, the SOS global transcriptional repressor (Little et al., 1980; Horii et al., 1981). LexA repressor cleavage de-represses expression of >40 genes comprising the network, including the *dinB*, *recA*, and *lexA* genes themselves (Fernandez De Henestrosa et al., 2000; Courcelle et al., 2001; Khil and Camerini-Otero, 2002; Friedberg et al., 2006). RecA, a multifunctional protein, plays essential roles in maintaining genomic integrity; its activities include mediating strand exchange in homologous recombination (Chintapalli et al., 2013), coordinating the access of DNA polymerases at the replication fork (Indiani et al., 2013), and slowing down DNA replication upon DNA damage (Tan et al., 2015).

DNA polymerase IV, an evolutionarily conserved error-prone DNA polymerase, is among the very first proteins upregulated during the SOS response as a consequence of the weak affinity between the LexA repressor and its binding site on the *dinB* promoter (Fernandez De Henestrosa et al., 2000; Courcelle et al., 2001). DinB is well-known for its ability to catalyze TLS, during which it bypasses specific DNA lesions on the template strand that would otherwise result in lethal replication fork stalling (Goodman, 2002; Friedberg et al., 2006; Jarosz et al., 2009). TLS is enabled by DinB’s open active site, which allows the enzyme to accommodate DNA lesions, though at the forfeiture of reliable geometric basepair checking (Yang, 2003; Jarosz et al., 2009). DinB is thus error-prone [at least a 10-fold higher mutation frequency on undamaged DNA compared to high fidelity DNA polymerase III’s (Tang et al., 2000)], possessing a mutational signature of -1 frameshifts and specific base pair substitutions (Kim et al., 2001; Nohmi, 2006). Accordingly, DinB activity has been associated with acquisition of bacterial antibiotic resistance (Bull et al., 2001; Cirz et al., 2005).

Despite LexA regulation, the basal level of DinB in *E. coli* is still relatively high [∼250 nM (Kim et al., 2001)] when compared to other DNA polymerases in the cell [e.g., 40 nM for the catalytic alpha subunit of DNA polymerase III (Pol IIIα) (Maki et al., 1985)]. As a consequence, other means of regulating DinB activity are physiologically important. One of these is the formation of a higher-order protein complex with RecA and a dimer of full-length UmuD, an accessory subunit. While in this complex, the enzyme generates fewer -1 frameshift mutations and has increased catalytic activity on properly aligned templates (Godoy et al., 2007). Structural docking based on peptide interaction data suggests that the binding of RecA and UmuD encloses the DinB active site. The interactions likely reduce DinB-mediated mutagenesis by restricting template looping that is required for frameshift events (Godoy et al., 2007). Recently, we showed that DinB also forms complexes *in vivo* exclusively with RecA (Cafarelli et al., 2013), and that this positively affects DinB fidelity (Cafarelli et al., 2014).

The other critical regulatory interaction made by DinB is with the beta processivity clamp, a key player of the cell’s replication machinery (Wagner et al., 2000). The beta clamp increases the processivity of various DNA polymerases (Wagner et al., 2000; Maor-Shoshani and Livneh, 2002; Vidal et al., 2004) and is partially responsible for coordinating polymerase switching at the replication fork (Kath et al., 2014). DinB’s affinity for the beta clamp is lower than that of Pol IIIα [K_D_ = 460 nM (Heltzel et al., 2012) and 108 nM (Heltzel et al., 2009) respectively]. It is possible that under non-DNA damaging conditions, this ∼4-fold difference contributes to preventing DinB from accessing the replication fork instead of Pol IIIα, though there is no direct evidence that speaks to this. However, upon DNA damage DinB is clearly the most abundant DNA polymerase in the *E. coli* cell at a concentration of approximately 2500 nM (Kim et al., 2001). Remarkably, in the DNA damage induced cell, the ratio of DinB:Pol IIIα is at approximately 60:1 (Maki et al., 1985; Kim et al., 2001). Given that the relative affinity of the beta clamp for DinB is only approximately four times lower than that for Pol IIIα and that DinB is in such excess in the cell, other mechanisms are likely necessary to prevent DinB synthesis on undamaged DNA.

Here, we report that DinB performs poor DNA synthesis with RNA primers and that this synthesis is further impeded upon interaction with RecA. The mechanism of this inhibition of synthesis is through a seemingly robust pause that is independent of template and lesion and is likely to result in abortive DNA synthesis when RNA is the primer. Poor synthesis of DinB using RNA primers might represent a way to prevent DNA synthesis by DinB when it is not needed. Our data provide novel insight into the mechanisms of regulation of error-prone DinB, which will, in turn, permit a deeper understanding of the relationship between DNA damage, mutagenesis, and genomic stability.

## Materials and Methods

### Strains, Plasmids, and Oligonucleotides

The strain TMCΔT: BL21-AI (Life Technologies, Carlsbad, CA, USA) *ΔdinB*, *ΔumuDC*, *ΔrecA*, was used for protein purification, and was constructed by P1 transduction (Thomason et al., 2007) using as a host the BL21-AI *ΔdinB, ΔumuDC* strain (Cafarelli et al., 2013). The construction of the RecA overproducing plasmid (pILRecA) is described below while the DinB overproducing plasmid (pDFJ1) has been previously published (Jarosz et al., 2006). The TMCΔT strain with the overproducing plasmids was grown in Luria broth medium with ampicillin [TMCΔT/pDFJ1 (Jarosz et al., 2006); 100 μg/mL] or kanamycin (TMCΔT/pILRecA; 35 μg/mL). Protein induction conditions are described below. All oligonucleotides used in this work are listed in Supplementary Table 1.

### Protein Purification

The native DinB overproducer plasmid, pDFJ1 (Jarosz et al., 2006), was introduced by transformation into the TMCΔT strain. Native DinB was overexpressed by autoinduction (Studier, 2005; Cafarelli et al., 2013) and cells were lysed using a cell homogenizer (Cafarelli et al., 2013). DinB was purified by ion exchange and hydrophobic interaction chromatography as previously published (Beuning et al., 2006). This will be referred to as naïve DinB in this report, since it has never been in contact with RecA.

The plasmid overproducing RecA was constructed as follows: the *recA* gene was amplified by PCR using the plasmid pCA24N (Konola et al., 1998) as template. The *recA* gene-containing amplicon was cloned into the pET His6 TEV LIC cloning vector (plasmid 29653; Addgene, Cambridge, MA, USA) by ligation independent cloning (LIC). PCR cycling conditions were based on the melting temperature of primer pairs. Primer sequences are listed in Supplementary Table 1. The resulting plasmid, pILRecA, was introduced by transformation into the TMCΔT strain. Cells were grown to saturation at 37°C in Luria broth medium with 35 μg/mL kanamycin and 0.05% (v/v) glucose. A 1 L culture of the same medium was inoculated with a 1:1,000 dilution from the saturated culture and grown at 37°C with agitation (250 rpm) until it reached an OD600 of 0.7. Protein overexpression was then induced by adding 0.05% (v/v) of L-arabinose. The culture was incubated at 20°C with agitation as before for approximately 12 h. Native naïve RecA (never in contact with DinB) was purified following a protocol previously described in Chen et al. (2008), but an approximate 1:15 (w/w, TEV protease/substrate) dilution of tobacco etch virus (TEV) protease (QB3 Macrolab, University of California, Berkeley, Berkeley, CA, USA) was used to cleave the His-tag from the RecA preparations. Purity of all proteins was determined by SDS-PAGE (Supplementary Figure 1).

### Primer Extension Assays

**FIGURE 1:**
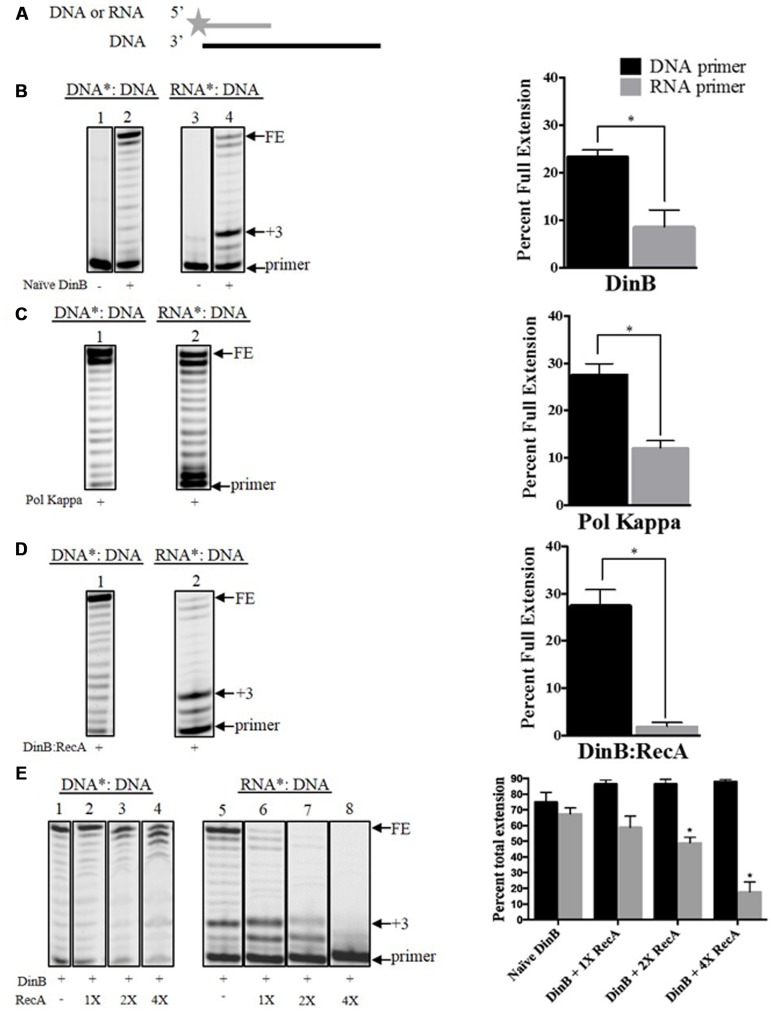
**Interaction with RecA further hinders DinB’s inherently poor RNA primer extension. (A)** Depiction of the primer extension assays carried out in the current work. The fluorescently labeled DNA or RNA primer is shown as a gray line with a star in the 5′ end. The DNA template is shown as a black straight line. The enzymes used are added to the reaction with the depicted substrate, which will extend from the primer. The primer extension assay products are separated in a denaturing acrylamide gel. Only extended products are observed under these conditions **(B)** Shown are the extension products resulting from the activity of 0.6 μM naïve DinB separated on a denaturing acrylamide gel or **(C)** DNA polymerase Kappa. Also shown is the quantification of the percent of full extension, calculated as indicated in Section “Materials and Methods.” These graphs show a twofold difference in DinB or Pol Kappa DNA primer extension compared to RNA primer extension. ^∗^ Indicated *p*-value < 0.05. **(D)** The DinB:RecA was formed by incubating the DinB and RecA proteins in a 1:1 molar ratio at room temperature for 1 h (see Materials and Methods). Extension products resulting from the activity of 0.6 μM DinB:RecA were quantified and show a significant reduction in full RNA primer extension as compared to naïve DinB. ^∗^ Indicated *p*-value < 0.05. **(E)** RecA and DinB were incubated, as in **(D)**, with increasing molar ratios. Higher concentrations of RecA significantly inhibit DinB’s total extension using RNA primers while having little effect on DinB’s total extension using DNA primers. ^∗^ Indicated *p*-value < 0.05 when comparing to extension by naïve DinB. All primer extension experiments were performed in triplicate. The quantification was carried out using extension products separated on the same gel. Images depict representative examples. Error bars indicate mean ± standard error. DNA^∗^: DNA, fluorescently labeled DNA primer annealed to the DNA template; RNA^∗^: DNA, fluorescently labeled RNA primer annealed to the DNA template; FE, fully extended primer band; +3, primer plus three nucleotide insertions band.

Primer extension assays were carried as previously described (Cafarelli et al., 2013) using Cy3 5′ labeled DNA or RNA primers annealed to various DNA templates (Supplementary Table 1 and **Figure 1A**). We used undamaged templates containing adenine (A) at the primer-template junction as well as a lesion-containing template with a 3-deaza-3-methyladenine (3d-meA) lesion at the same position (Supplementary Table 1). Reactions contained a mixture of 500 μM dNTPs (Takara, Otsu, Shiga, Japan), buffer and similar concentrations of the different DNA polymerases. For DinB:RecA reactions, naïve DinB and RecA were incubated in buffer SA [50 mM Hepes, 10% glycerol (v/v), 2 mM 2-mercaptoethanol, pH 7.5] at a 1:1 molar ratio at room temperature for 1 h before 0.6 μM were added to the primer extension reaction mix. Polymerase Kappa was a gracious gift of Janice Pata (Wadsworth Center, NYS Department of Health) and DNA polymerase I was obtained from New England Biolabs (Ipswich, MA, USA). The undamaged C3 DNA template (Supplementary Table 1) was used unless otherwise indicated. All reactions were performed in triplicate and direct comparisons were made only of samples separated in the same gel. Percent full extension was calculated as the fraction of the FE product divided by the total obtained from all products (**Figure 1**). Percent total extension was calculated as the fraction of extended products minus the primer divided by the total obtained from all products (**Figure 3**).

## Results

### Naïve DinB extends Poorly from an RNA Primer

To our knowledge there is no information published regarding DinB’s ability to perform DNA synthesis using an RNA primer. It is known that *in vitro* DinB considerably slows DNA replication by Pol IIIα specifically on the lagging strand of synthesis (Indiani et al., 2009) and can switch with Pol IIIα at the replication fork during DNA synthesis (Heltzel et al., 2009, 2012). Due to DinB’s high intracellular concentration [lowest levels: 250 nM (Kim et al., 2001), compared to 40 nM of Pol IIIα (Maki et al., 1985)], and the high availability of RNA primers to copy the lagging strand, we were interested in assessing DinB’s inherent ability to carry out DNA synthesis using RNA primers.

We first used naïve DinB, which has never been in contact with RecA, to measure extension of a fluorescently labeled DNA or RNA primer annealed to the same unlabeled DNA template (**Figure 1A**). The reactions conditions and enzyme concentrations were consistent for primer extension reactions involving DNA or RNA primers. The products of these reactions were then examined by denaturing gel electrophoresis. We did not observe a significant difference between DNA and RNA primer extension in experiments with the high fidelity DNA polymerase I (Supplementary Figure 2). In contrast, we find that naïve DinB extended significantly less from an RNA than from a DNA primer (**Figure 1B**). Remarkably, we find that this property is conserved in DNA Pol Kappa (**Figure 1C**), the human homolog of DinB. We also noted a prominent band at the third nucleotide insertion (+3 site, see +3 in **Figure 1B**) only in DinB’s RNA primer extension products. The synthesis beyond this third addition was significantly diminished as compared to synthesis from a DNA primer (compare bands above +3 in lanes 2 and 4, **Figure 1B**).

### Interaction with RecA Further Hinders DinB’s Synthesis Using RNA Primers

We have previously shown that DinB and RecA co-purify (Cafarelli et al., 2013) and that their interaction enhances DinB’s fidelity (Cafarelli et al., 2014). We investigated whether RecA interaction also affects DinB’s extension of RNA primers. We find that, remarkably, DinB:RecA’s primer preference (i.e., difference between full extension using DNA or RNA primers) is significantly higher than that of naïve DinB. While RecA may be capable of binding ssDNA that is present in the template, we have previously shown that RecA in a 1:1 molar ratio with DinB does not inhibit DinB activity with a DNA primer [(Cafarelli et al., 2014), also compare lane 2 in **Figure 1B** to lane 1 in **Figure 1D**)]. Thus, it is unlikely that RecA’s ssDNA binding is responsible for reduced DinB activity in the RNA primer experiments (**Figure 1D**). Notably, the prominent band at the third nucleotide insertion that is observed in the naïve DinB extension assays was also seen here (compare +3: lane 4 in **Figure 1B** and lane 2 **Figure 1D**).

We noticed that mixing DinB and RecA in a 1:1 molar ratio has a significant effect on DinB’s full extension (compare FE band in **Figure 1B**, lane 4 and **Figure 1D**, lane 2). However, DinB interaction with RecA had little effect on the addition of the first three nucleotides (compare +3 band and below in **Figure 1B**, lane 4 and **Figure 1D**, lane 2), which accounted for most of the extension products observed in these reactions. As the uninduced RecA concentration in *E. coli* is approximately fourfold higher than that of DinB (Salles and Paoletti, 1983; Kim et al., 2001), we sought to determine how higher RecA concentration would alter DinB’s extension. To account for the first three-nucleotide insertions, we compared in these experiments the percent total extension (i.e., the quantification of all extension products divided by the quantification of the extension products plus the primer). Interestingly, increasing concentrations of RecA significantly inhibits DinB’s total extension using RNA primers, while having no significant effect on its extension using DNA primers (**Figure 1E**). These data indicate that interaction with RecA further inhibits DinB’s already poor ability to synthesize DNA using RNA primers.

### DinB’s Poor RNA Primer Extension Is Not Due to RNA Primer Degradation, Poor Annealing, or RNase Contamination

**FIGURE 2:**
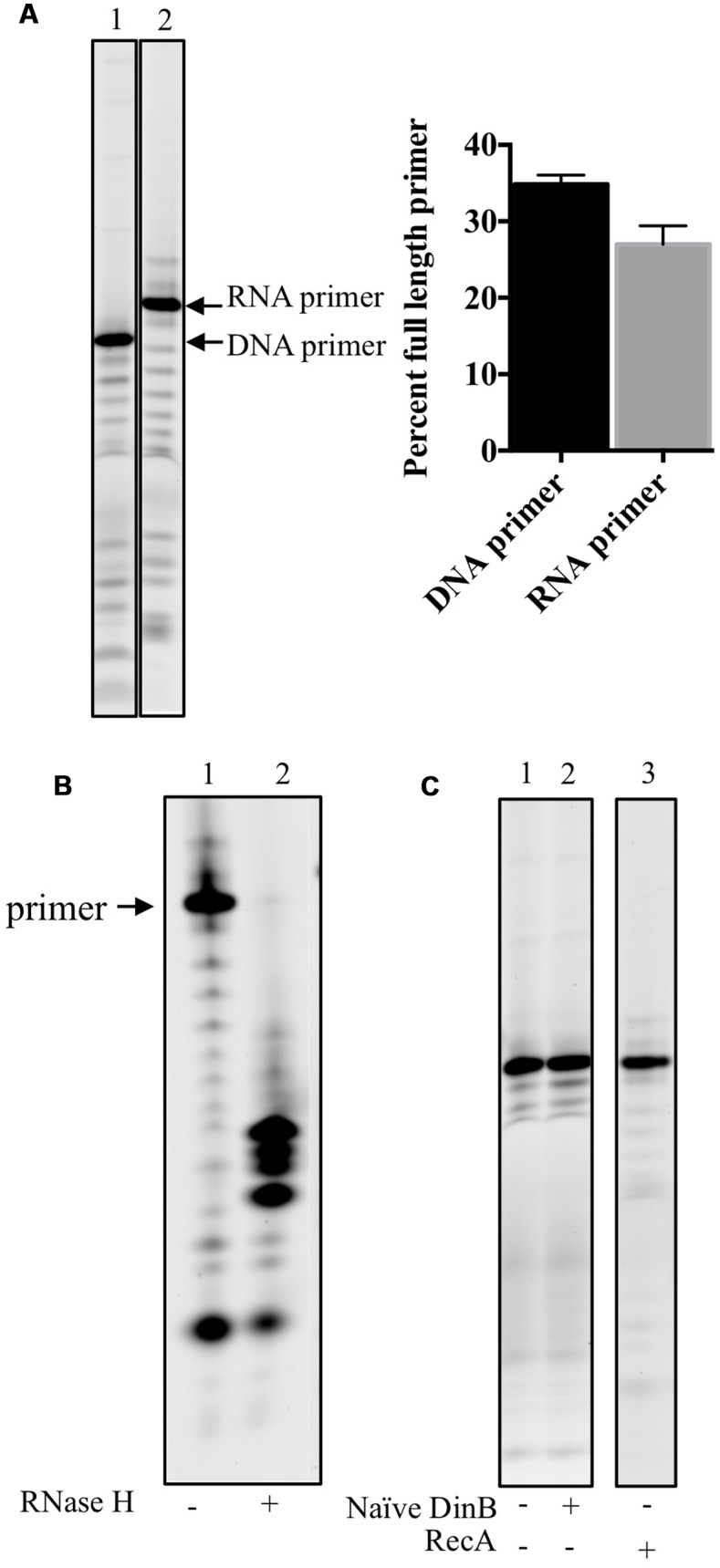
**RNA primers used in this study are stable, anneal adequately to the DNA template and no RNase contamination is detected in stocks of purified proteins. (A)** DNA and RNA primers separated on acrylamide gels are quantified to determine the stability of the labeled primers. Percent full-length primer was calculated as the percent of total band intensity found in the full-length band (labeled RNA or DNA primer). As shown in the graph in the right hand side of the gel image, there are comparable levels of full-length primers indicating they are equally stable in the assay conditions. Error bars indicate mean ± standard error. **(B)** RNase H digests only RNA-DNA hybrid molecules, therefore the RNA: DNA primer template mixes were left untreated (-) or treated (+) with RNase H. The results of the experiments show that most of the RNA primer is annealed to the DNA template as only a negligible band of the full-length primer is observed in the RNase H digestion of RNA primer annealed to DNA template. The products of the digestion are observed in the (+) lane underneath of what it would have been the full-length primer band. **(C)** To test whether the protein stocks used had contaminating RNase activity, 0.6 μM of naïve DinB or RecA protein is incubated with the labeled RNA primer annealed to DNA template in the absence of dNTPs. No degradation of the primer was observed as there is no difference between the (-), no protein, lanes are the same as the (+) lanes. These data indicate that the respective protein stocks are not contaminated with an RNase. All experiments were repeated three times and similar results are observed every time. Images are from representative gels. The products shown in the lane of RecA (+), right hands side of C, were separated on a different gel than the one shown for naïve DinB.

To ensure that DinB poor RNA primer extension was not due to a difference in concentration or degradation of the RNA primers, the un-annealed DNA or RNA primers were separated using denaturing gel electrophoresis (**Figure 2A**). The full-length band for either primer was quantified as the percent of full-length primer in relation to lower bands and found to be comparable (graph in **Figure 2A**).

We next sought to examine another possible technical explanation for the data in **Figure 1**: that the RNA primer is not efficiently annealing to the DNA template. Therefore, RNase H was used to digest the RNA primer annealed to the DNA template. Since RNase H only digests RNA-DNA hybrids, inefficient annealing would result in undigested RNA primer (similar to the undigested control, lane 1 in **Figure 2B**). However, we find only smaller fragments (lane 2 in **Figure 2B**) and negligible evidence of the full-length RNA primer after RNase H digestion, indicating that most of the RNA primer had successfully annealed to the DNA primer.

Finally, as an RNase contamination of the purified protein preparation could possibly account for poor RNA primer extension, we incubated purified naïve DinB or RecA with the RNA primer annealed to DNA template in the absence of dNTPs and found no visible RNA primer degradation, suggesting this possibility is unlikely (**Figure 2C**).

These data led us to conclude that naïve DinB’s poor RNA primer extension (**Figures 1B,D**) arises from *bona fide* inefficient synthesis from an RNA primer.

### DinB Extension from an RNA Primer is Reduced by Addition of Unlabeled DNA primer:template with Little Effect on DinB Extension from a DNA Primer

**FIGURE 3:**
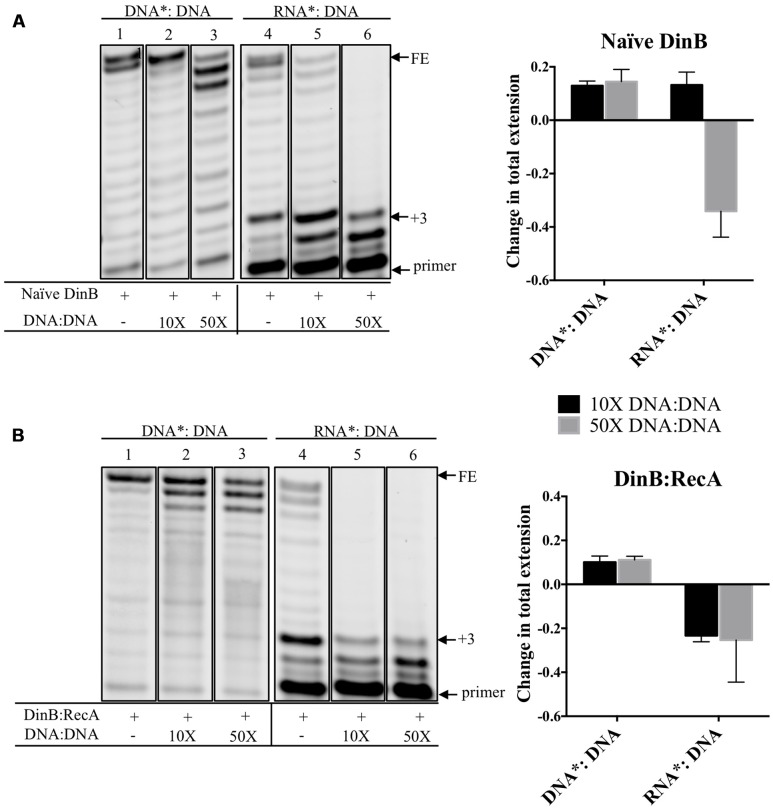
**Competition experiments using unlabeled DNA primer:template show that the extension from a labeled RNA primer is reduced for naïve DinB or DinB:RecA with little effect on the extension from a DNA primer. (A)** Naïve DinB DNA primer extension assays are not competed by a 10 or 50-fold excess of unlabeled DNA: DNA primer template as shown in the first three lanes. In contrast the RNA primer extension assays show competition with a 50-fold excess of the unlabeled DNA: DNA primer template. The quantification of the change in total extension is shown in the right hand side graph. The bars shown above zero indicate no change while the bars below zero indicate competition. **(B)** DinB:RecA was formed as indicated in **Figure 1** legend. DinB:RecA DNA primer extension activity was unchanged by competing 10 or 50-fold excess of the DNA: DNA primer template. This is shown in the first three lanes. The competition by the DNA: DNA on the RNA primer extension activity of DinB:RecA is evident with both a 10 or 50-fold excess. Quantification of total extension is described in Section “Materials and Methods.” All experiments were performed in triplicate and comparisons were made of products separated on the same gel. Quantification is shown in the right hand side of the gel images. Images are of representative gels. Error bars indicate mean ± standard error. DNA^∗^: DNA, fluorescently labeled DNA primer annealed to DNA template; RNA^∗^: DNA, fluorescently labeled RNA primer annealed to DNA template; DNA: DNA, unlabeled DNA primer annealed to unlabeled DNA template; FE, fully extended primer band; +3, primer plus three nucleotide insertions band.

We measured the effect of adding increasing concentrations of unlabeled DNA: DNA on DinB extension from a labeled RNA primer annealed to the same DNA template (RNA^∗^: DNA). We predicted that the unlabeled DNA: DNA should decrease the extension from the RNA^∗^: DNA due to DinB’s preference for DNA primers, while having less of an effect on DinB extension from a labeled DNA primer annealed to a DNA template (DNA^∗^: DNA). Quantification of the data gathered from separating the extension products in a denaturing gel (lanes 4–6 in **Figure 3A**) indicate that the addition of 50-fold higher concentration of DNA: DNA reduced naïve DinB’s total extension from the RNA^∗^: DNA by ∼34% (graph in **Figure 3A**). As predicted, the addition of DNA: DNA had little effect on the total extension of DinB on DNA^∗^: DNA (lanes 1–3 in **Figure 3A**).

Because there is a larger difference in DinB:RecA’s ability to synthesize from DNA versus RNA primers compared to naïve DinB, we predicted that smaller concentrations of competing DNA: DNA would be required to reduce DinB:RecA’s extension on RNA^∗^: DNA. Notably, addition of only 10-fold higher concentration of DNA: DNA reduced total extension of the RNA^∗^: DNA by ∼23% (graph in **Figure 3B**), though total extension from DNA^∗^: DNA is similar to that observed and quantified in naïve DinB (compare lanes 1–3 in **Figure 3A** to lanes 1–3 in **Figure 3B**). These results support the assertion that DinB:RecA synthesizes using RNA primers even more poorly than naïve DinB and suggests that there might be a change in the DinB active site when interacting with RecA. This is consistent with our previous findings that interaction with RecA affects DinB function (Godoy et al., 2007; Cafarelli et al., 2013, 2014).

### Naïve DinB and DinB:RecA Pausing on an RNA Primer is Independent of Template Sequence and Lesion

**FIGURE 4:**
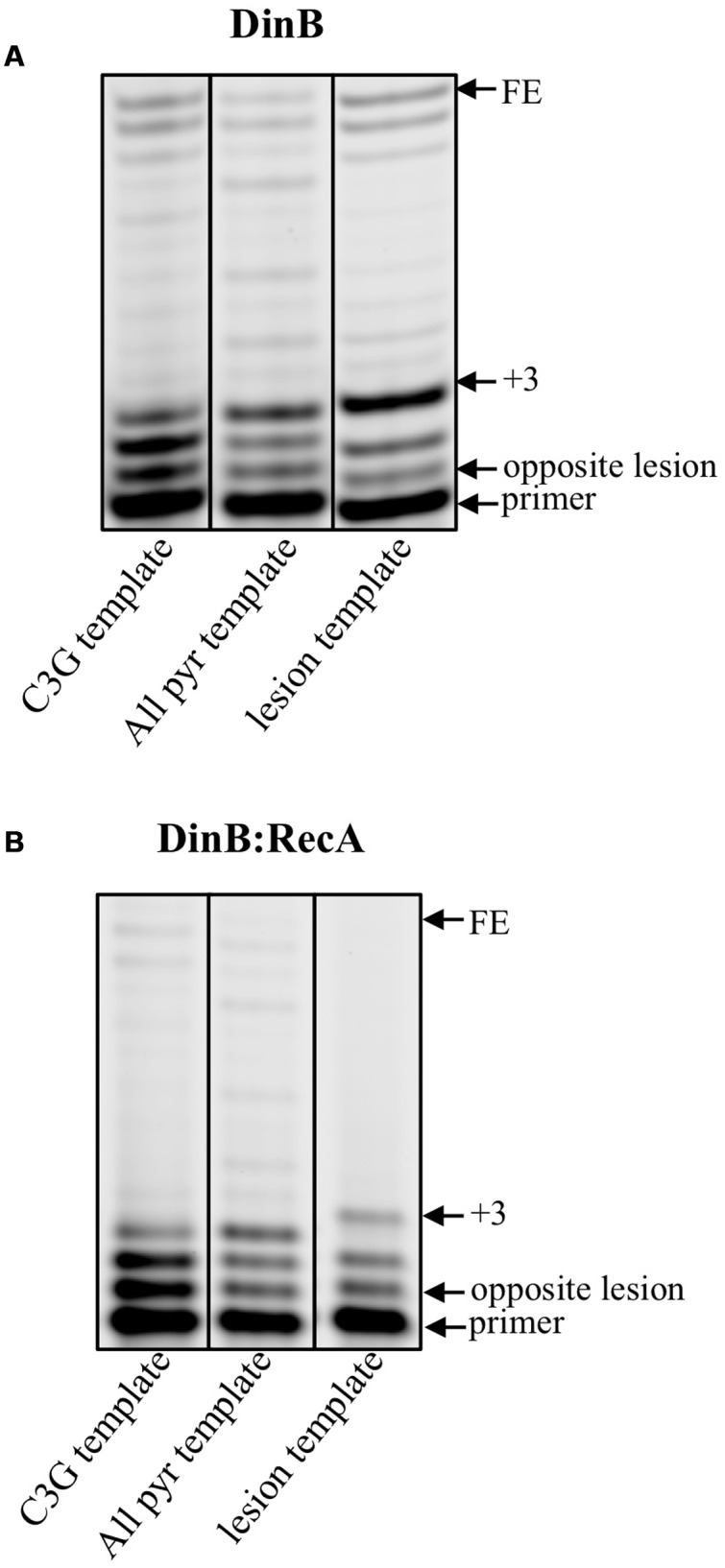
**The accumulation of a unique +3 extension product only when an RNA primer is used for extension assays is independent of template sequence or lesion. (A)** Naïve DinB activity was measured by extension of a fluorescently labeled RNA primer annealed to an unlabeled DNA template with the different sequences or with a DNA lesion right at the primer-template junction (3-deaza-3-methyl-adenine). Gel images depicting the RNA extension products clearly show accumulation of a +3 product regardless of the template sequence or lesion. **(B)** DinB:RecA was formed as indicated in **Figure 1** legend. Then the DinB:RecA activity was measured in the manner described in A. The gel images show the presence of the +3 accumulation product occurring regardless of template sequence or lesion. Experiments were performed in triplicate with independent protein preparations with similar results. Images depict representative examples separated on the same gel. FE, fully extended primer band; +3, primer plus three-nucleotide insertions product; C3G, All pyr, and lesion templates are described in Supplementary Table 1.

From the experiments described above, we know that when both naïve DinB and DinB:RecA (1:1) extend from an RNA primer there is a clear accumulation of a +3 insertion product (lane 4 **Figure 1B** and lane 2 in **Figure 1D**). This is not observed on the DNA primer (lane 2 **Figure 1B** and lane 1 in **Figure 1D**). To rule out the possibility that this accumulation is due to a template sequence effect, we changed the sequence of the template at the nucleotides opposite from the +3 insertion site from C to G (C3G DNA template, Supplementary Table 1). This sequence change had no effect on the accumulation of the +3 insertion product for either naïve DinB (C3G template, **Figure 4A**) or DinB:RecA (C3G template, **Figure 4B**). We also changed every purine in the template sequence to a pyrimidine (All pyr DNA template, Supplementary Table 1) and we observe no effect on the +3 insertion product regardless of whether we used naïve DinB (All pyr template, **Figure 4A**) or DinB:RecA (All pyr template, **Figure 4B**). These data indicate that the +3 insertion product accumulation is independent of template sequence.

Due to DinB’s activity as a TLS polymerase, we determined whether both naïve DinB and DinB:RecA have the ability to bypass a lesion from a template that contained 3d-meA at the primer:template junction (Supplementary Table 1). We observed that there is lesion bypass, but the accumulation of the +3 insertion product for naïve DinB or for DinB:RecA (lesion template, **Figures 4A,B** respectively) occurs regardless of the template lesion indicating that the +3 pause is lesion independent.

The +3 insertion pause is reminiscent of one previously described for the derivative DinB(Y79L) (Jarosz et al., 2009) in experiments of lesion bypass. This pause was interpreted as being part of an important regulatory mechanism for TLS with cell survival consequences; strains with the plasmid-borne DinB(Y79L) are susceptible to DNA damage generated by the antibiotic nitrofurazone (Jarosz et al., 2009).

It is possible that during RNA primer extension DinB’s active site resembles that of DinB(Y79L), in which tyrosine (Y) 79 has been changed to leucine (L), resulting in a similar pause. Pausing and aborted DNA synthesis, like that predicted in the DinB(Y79L) variant (Jarosz et al., 2009), might represent a mechanism by which DinB is prevented from synthesizing on the lagging strand of DNA synthesis. This preferential activity suits the role of DinB as a specialized DNA polymerase with DNA damage tolerance activities.

## Discussion

DinB, the most evolutionarily conserved TLS DNA polymerase (Ohmori et al., 2001), is prone to causing mutations (Tang et al., 2000). Therefore, *dinB* gene expression (Fernandez De Henestrosa et al., 2000; Courcelle et al., 2001; Khil and Camerini-Otero, 2002; Friedberg et al., 2006) and DinB activity (Godoy et al., 2007; Cafarelli et al., 2013, 2014) are tightly regulated. In particular, DinB activity is governed by protein–protein interaction with RecA and a dimer of UmuD (Godoy et al., 2007). The binding of these interacting partners visibly alters the mutagenic potential of DinB *in vivo* and *in vitro* (Godoy et al., 2007; Cafarelli et al., 2013, 2014). We have also established that RecA binds to DinB prior to UmuD and proposed that DinB interacting with RecA may occur more frequently than the DinB•RecA•UmuD_2_ complex *in vivo* (Cafarelli et al., 2013). We have also shown that RecA interaction positively alters DinB fidelity (Cafarelli et al., 2014), which we presumed is due to a change in the DinB active site conformation upon binding to RecA.

We report here that DinB poorly extends from RNA primers (**Figure 1**). During non-DNA damaging conditions the affinity of the beta clamp for DinB, though lower than the affinity for DNA Pol IIIα (Heltzel et al., 2009, 2012), may be sufficient to prevent DinB from accessing the forming replication fork at RNA primers, even though DinB is present at a significantly higher concentration (Maki et al., 1985; Kim et al., 2001). We hypothesize that the drastically increased concentration of DinB upon DNA damage would overwhelm the affinity for the beta clamp allowing DinB access to the lagging strand. In fact, upon SOS induction, the dramatically higher concentration of DinB (Kim et al., 2001) slows the rate of replication by Pol IIIα (Tan et al., 2015), likely occurring at the lagging strand, suggesting that the elevated DinB intracellular concentration overwhelms the selectivity of the beta clamp and allows DinB to access the replication fork. Indeed, previous studies have shown that DinB slows replication by Pol IIIα specifically on the lagging strand (Indiani et al., 2009). We find that DinB:RecA synthesizes poorly using RNA primers compared to DNA primers beyond the third nucleotide insertion and that this poor synthesis is independent of template sequence or of a lesion (**Figure 4**). DinB:RecA (or DinB) would access the replication fork on the lagging strand, but would not efficiently synthesize from RNA primers providing a mechanism to slow down replication with a lowered mutagenic cost.

The pausing observed in DinB (or DinB:RecA) using an RNA primer (**Figures 1**, **3**, **4**) resembles that caused by a DinB variant, DinB(Y79L), which also pauses exactly three nucleotides after a template lesion (Jarosz et al., 2009). In the native DinB protein, the Y79 residue interacts with another aromatic residue, phenylalanine (F) 13, which in turn interacts with the incoming nucleotide (Nevin et al., 2015). During TLS, the loss of the large Y aromatic residue in DinB(Y79L) is hypothesized to cause a conformational change of the active site, which leads to the DinB pausing three nucleotides after encounter of a lesion (Jarosz et al., 2009; Nevin et al., 2015). It is remarkable that the use of an RNA primer results in a similar DinB pausing (**Figures 1**, **3**, **4**), suggesting the RNA primer may induce a similar active site conformation as that of DinB(Y79L). Moreover, the DinB interaction with RecA would stabilize this change. The pausing observed in the DinB(Y79L) variant is hypothesized to be part of an abortive TLS mechanism, which would lead to a futile cycle of DinB pausing and DNA polymerase III excision repair (Jarosz et al., 2009). It is possible that native DinB undergoes a similar cycle of abortive synthesis with an RNA primer. This mechanism could account for the sickness and lethality that has been observed upon DinB overproduction (Benson et al., 2014).

The data reported here identify a pausing in DinB’s DNA synthesis while using an RNA primer. This novel finding represents an important potential mechanism for preventing the high intracellular concentration of DinB from inducing unnecessary mutagenesis.

## Author Contributions

TT: designed and performed experiments, analyzed data, and wrote paper; IL: performed and designed RecA protein purification experiments and analyzed data; VB: performed initial RNA primer extension experiments; TC: designed initial experiments and analyzed data; VG: designed experiments, analyzed data and wrote paper.

## Conflict of Interest Statement

The authors declare that the research was conducted in the absence of any commercial or financial relationships that could be construed as a potential conflict of interest.

## Abbreviations

All pyr template: DNA template with in which all purines are mutated to pyrimidines
Naïve DinB: DinB that has not been in contact with RecA
DinB:RecA: a mix of proteins containing a 1:1 molar ratio of DinB to RecA
DinB: DNA polymerase IV
DNA^∗^:DNA: fluorescently labeled DNA primer annealed to a DNA template
DNA:DNA: unlabeled DNA primer annealed to a DNA template
C3G template: DNA template with a C-to-G mutation across from the third nucleotide insertion site
FE: fully extended primer
Lesion template: DNA template with a 3-deaza-3-methyladenine lesion across from the first nucleotide insertion site
RNA^∗^:DNA: fluorescently labeled RNA primer annealed to a DNA template
Pol IIIα: alpha subunit of DNA polymerase III
TLS: translesion synthesis
3d-meA: 3-deaza-3-methyladenine
+3: primer with three nucleotide insertions
