# Supplementary material for: RNA Primer Extension Hinders DNA Synthesis by Escherichia coli Mutagenic DNA Polymerase IV
Source: Front Microbiol. 2017 Mar 1;8:288. doi: 10.3389/fmicb.2017.00288 (PMC5331060; doi:10.3389/fmicb.2017.00288)
Supplement: Supplementary file 1 [file Table_1.PDF]

**Supplementary Table 1: Oligonucleotides used in this study**

| Nucleotide Sequence (5’ to 3’) | Purpose |
|---|---|
| TACTTCCAATCCAATGCAATGGCTATCGACGA | *recA* TEV LIC forward primer |
| TTATCCACTTCCAATGTTATTATTAAAAATCTTCGTTAGTTT | *recA* TEV LIC reverse primer |
| /HEX/CAC TGC AGA CTC TAA A | Cy3 labeled DNA primer for extension assays |
| /HEX/CAC UGC AGA CUC UAA A | Cy3 labeled RNA primer for extension assays |
| GCTCGTCAGA**C**GATTTAGAGTCTGCAGTG | Undamaged C3 DNA template |
| GCTCGTCAGA**G**GATTTAGAGTCTGCAGTG | Undamaged C3G DNA template |
| GGAGGAGAGA**G**GATTTAGAGTCTGCAGTG | Undamaged all pyr DNA template |
| GCTCGTCAGACG/3d-meA/TTTAGAGTCTGCAGTG | Lesion-containing DNA template |
