# Supplementary material for: RNA Primer Extension Hinders DNA Synthesis by Escherichia coli Mutagenic DNA Polymerase IV
Source: Front Microbiol. 2017 Mar 1;8:288. doi: 10.3389/fmicb.2017.00288 (PMC5331060; doi:10.3389/fmicb.2017.00288)
Supplement: Supplementary file 2 [file Image_1.PDF]

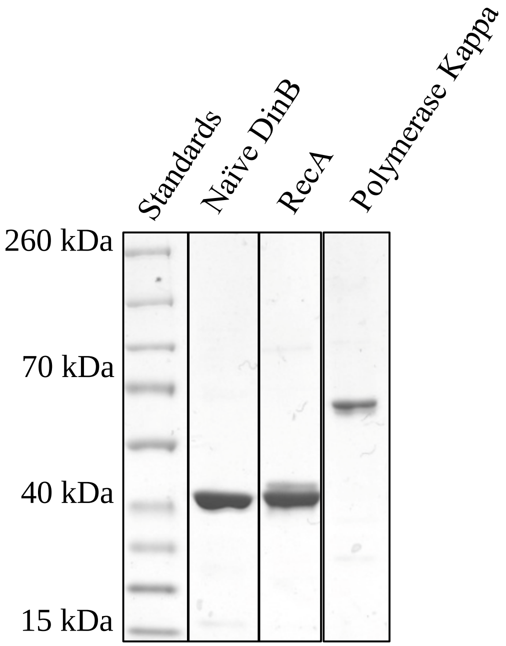

Supplementary Figure 1. Purified proteins used in this study. All proteins were analyzed by SDS-PAGE and stained with Coomassie Brilliant Blue. Standards, Spectra Broad Range Protein Standards. Polymerase Kappa obtained from Janice Pata (Wadsworth Center, NYS Department of Health).
