# Supplementary material for: RNA Primer Extension Hinders DNA Synthesis by Escherichia coli Mutagenic DNA Polymerase IV
Source: Front Microbiol. 2017 Mar 1;8:288. doi: 10.3389/fmicb.2017.00288 (PMC5331060; doi:10.3389/fmicb.2017.00288)
Supplement: Supplementary file 3 [file Image_2.PDF]

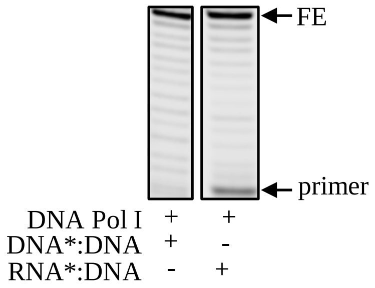

Supplementary Figure 2: DNA primer preference is not conserved in DNA Pol I. High fidelity DNA polymerase I (125 nM) does not have a difference in primer extension between DNA and RNA primers. Experiments performed as in Figure 1. DNA*: DNA, fluorescently labeled DNA primer annealed to DNA template; RNA*: DNA, fluorescently labeled RNA primer annealed to DNA template; FE, fully extended primer band.
